# Shape–Trait Consistency: The Matching Effect of Consumer Power State and Shape Preference

**DOI:** 10.3389/fpsyg.2021.615647

**Published:** 2021-09-30

**Authors:** Feng Yao, Xiaotong Jin, Banggang Wu, Taiyang Zhao, Tiannv Ma

**Affiliations:** ^1^Department of Marketing, Business School, Jilin University, Changchun, China; ^2^Department of Marketing, Business School, Sichuan University, Chengdu, China; ^3^Department of Psychology, School of Philosophy and Sociology, Jilin University, Changchun, China; ^4^Department of Business Administration, School of Management, Changchun University, Changchun, China

**Keywords:** power state, shape preference, competence, warmth, product design

## Abstract

Angular and rounded shapes are two important visual elements widely used in the design of product shapes and brand logos. By introducing the power state, a psychological variable that is inherently relevant to consumers' product choices, brand preferences, and decision-making, we propose that consumers' power state influences their shape preference. Specifically, compared to low-power consumers, high-power consumers respond more positively to angular as opposed to rounded shapes, because the angular shape facilitates the expression of competence (as opposed to warmth). Through four studies, we provide consistent support for our main predictions as well as the underlying processes. Studies 1 and 2 demonstrate that consumers experiencing higher power are more likely to prefer an angular shape over a rounded shape than those experiencing lower power through different research methods, research objects, and experimental materials. On this basis, studies 3 and 4 further explore the mechanisms underlying the observed effects. These findings contribute to sensory marketing and power research and provide important implications for visual design and advertisement development.

## Introduction

Shape design is an important marketing element in product and brand logo design. Different shape designs communicate different product and brand connotations and also attract consumers with different psychological characteristics. Exploring consumers' shape preferences can not only help in understanding the psychological demands of consumers but also to accurately provide consumers with suitable products. The sense of power, as a basic psychological variable, has an important influence on consumers' cognition, emotion, and behavior. Therefore, when faced with different shape designs, how do consumers with different power states choose?

Power is defined as “asymmetric control over valued resources in a social relationship” (Galinsky et al., 2008, 2015). Previous research has shown the power state has an important impact on a variety of consumer behaviors (Jin and Huang, 2018). However, existing research on the power state in consumer behavior areas mainly focuses on consumer persuasion (Dubois et al., 2016; Jin and Huang, 2018), compensatory consumption (Dubois et al., 2012), and behavior related to money and price (Garbinsky et al., 2014), which rarely provide direct guidance information for product design. Therefore, this study seeks to determine the kind of product shape that can better meet the preferences of consumers in different power states. Thus, it provides guidance and suggestions for product design.

Recently, angular and rounded shapes as important visual elements in product design have received increased attention from scholars and marketers. For example, Vallen et al. (2018) demonstrated that salespersons are more likely to recommend round (vs. angular) shaped products to heavier (vs. thinner) customers. Research on product selection suggests that in the process of product selection, people will seek the congruence between self-concept and product impression (i.e., self-image congruence; Sirgy, 1982; Sirgy et al., 2004). Previous studies revealed that individuals with high or low power states have different self-concepts (i.e., how individuals regard themselves) in many aspects. For example, compared with low-power consumers, high-power consumers are more confident in themselves, more creative, and have higher executive functioning (Galinsky et al., 2015).

Therefore, consumers with different power states would choose products that match their personal characteristics. According to the agentic–communal model of power, consumers experiencing a psychological state of high power have higher demand for competence. In contrast, consumers experiencing low-power state have higher demand for warmth (Dubois et al., 2016), which parallels the characteristics of angular and rounded shapes. Specifically, the angular shape expresses competence, while the rounded shape primarily expresses warmth. Therefore, when making consumption choices related to shape, high-power consumers prefer an angular shape with competence perception, while low-power consumers prefer rounded shapes with warmth perception.

## Theoretical Framework

### Power and Psychological Propensities

Psychological propensities of power refer to inclinations or tendencies naturally triggered by the psychological experience of power (Rucker et al., 2012). It is a behavioral tendency requiring little to no cognitive intervention. For example, power enhances people's action tendencies (Galinsky et al., 2003), making people more confident (Brinol et al., 2007). Similarly, power states affect consumers' psychological preferences for objective things (Kim and McGill, 2011).

Drawing on the seminal work of Bakan (1966), Rucker et al. (2012) proposed an agentic-communal model of power, suggesting the power state affects individuals' agentic-communal orientation and further affects individuals' thoughts and behaviors. Based on the agentic-communal model, high power tends to foster an agentic orientation where the self is viewed as more important and valued, and pursues independence and self-struggle more freely, relying less on others. Therefore, compared with low-power consumers, high-power consumers pay more attention to their own capabilities. In contrast, low power tends to foster a communal orientation where others are viewed as more important and valued (Rucker et al., 2011) and has a stronger group tendency, paying more attention to the needs of others. Due to their lack of sufficient capabilities and resources, low-power individuals often need to rely on others to achieve self-worth. Therefore, compared with high-power individuals, low-power individuals pay more attention to the connection with members of the surrounding groups and have a stronger appeal for warmth.

Meanwhile, according to the psychological tendency theory of power, individuals prefer to choose products that match their own characteristics (Galinsky et al., 2015). Thus, high-power individuals are more inclined to choose options related to competence. As one example, Rucker and Galinsky (2009) proposed that compared with low-power consumers, high-power consumers respond more positively to persuasive appeals that emphasize the quality and performance of products. On the contrary, the behavior and consumption choices of low-power individuals are more likely to be related to warmth. For example, low-power (vs. high-power) individuals are more willing to donate (Roux et al., 2012). Based on the above inferences, high-power consumers demonstrate they have a higher demand for competence, while low-power consumers have a higher demand for warmth. Dubois et al. (2016) also confirmed this view. In their research, they found high-power individuals pay more attention and are more easily persuaded by information related to competence; on the contrary, individuals with low power pay more attention and are more easily persuaded by information related to warmth. The existing research on power state and perceived competence and warmth mainly focuses on persuasion, but current research breaks through this limitation and introduces it into the field of sensory marketing to explore the relationship between power state and shape preference and its explanation mechanism. Meanwhile, through empirical test, it provides direct empirical evidence for the relationship between power state and perceived competence and warmth.

### Visual Shape and Stereotype Content Model

Angular and rounded are typical representatives of shape in visual design (Vallen et al., 2018; Yang and Chen, 2019). Angular shapes (e.g., rectangles, triangles, etc.) are composed of straight lines, planes, and sharp corners, while rounded shapes (e.g., circles, ellipses, etc.) are composed of smooth curves and arcs. The difference between rounded and angular shapes is not only reflected in the composition of the elements but also the symbolic meanings they hold (Arnheim, 1974). Different shapes bring different perceptive characteristics to consumers, which play an important role in consumers' value judgment and decision-making (Zhang et al., 2006; Zhu and Argo, 2013; Jiang et al., 2016). For example, Lieven et al. (2015) show that the shape of a brand logo will affect consumers' perception of brand “personalities.” Specifically, an angular shape will enhance the perception of the masculinity of brands, whereas rounded shapes will enhance the perception of the femininity of brands. Zhu and Argo (2013) demonstrated that in the negotiation process, an angular seating arrangement triggers a situational need to be unique, and individuals seated in such a setting respond more favorably to a self-oriented persuasive appeal, while a rounded seating arrangement triggers a situational need to belong and respond more favorably to a family-oriented persuasive appeal.

According to the stereotype content model (SCM; Fiske et al., 2007), individuals evaluate the target object through two fundamental dimensions of social judgments: competence and warmth. Warmth embodies traits of being friendly, trustworthy, and kindness, whereas competence is associated with traits of being competent, efficient, and skillful (Fiske et al., 2002; Aaker et al., 2010). The evaluation of the competence and warmth for the target object will further trigger the individuals' positive or negative affect, and then drive their consumption behavior. In the current study, we introduce competence and warmth into the expression of shape “personality,” recognizing the angular and rounded shape from the perspective of competence and warmth.

Prior studies on emotional expressions have revealed that people tend to use curved lines to express emotional states of gentleness, happiness, and quietness, while angled lines are used to express serious, harsh, and vigorous emotions (Lundholm, 1921; Poffenberger and Barrows, 1924). Jiang et al. (2016) also proposed that an angular brand logo leads to a sense of hardness and durability, while a rounded brand logo triggers softness and comfort perception to consumers. Therefore, based on the above research, we infer that compared to the rounded shape, the angular shape is superior to the perception of competence, while the rounded shape is superior in terms of the perception of warmth. This fully reflects the real-life symbolic meaning of the shapes. In work situations or sales services, for example, an angular face gives people a sense of seriousness and capability (competence), whereas a rounded face makes people looks gentler and friendlier (warmth).

In summary, the competence and warmth embodied by angular and rounded shapes are consistent with the appeal of high- and low-power consumers. Therefore, in the research on visual marketing related to shapes, it is proposed that the power state affects consumers' shape preference, and this influence is explained by the mediating effect of competence and warmth. Specifically, high-power individuals have higher demands for competence, so they tend to choose angular shapes that reflect competence characteristics, while low-power individuals have higher demands for warmth, so they prefer rounded shapes that reflect warmth. Accordingly, we propose the following hypotheses:

**Hypothesis 1**: Consumers experiencing higher power are more likely to prefer an angular shape over a rounded shape than consumers experiencing lower power.
**Hypothesis 2**: Perceived competence and warmth play a mediating role between power state and shape preference.

The above inferences about the mediating effect put forward the mechanisms underlying perceived competence and warmth. However, according to existing research theories, we also try to rule out two possible alternative explanations: conflict resolution types and need for uniqueness. The literature regarding angular and rounded shapes suggests angular shapes tend to generate confrontational associations, and rounded shapes tend to generate compromise associations (Zhang et al., 2006). According to the approach-inhibition theory of power (Keltner et al., 2003), having power activates consumers' approach-related tendencies, whereas lacking power activates consumers' inhibition-related tendencies. Therefore, conflict resolution types may be the mechanisms underlying the influence of power state on shape preference. Specifically, consumers with high power have a higher motivation to approach behavior, so they tend to be more confrontational, and thus prefer an angular shape. On the contrary, low-power consumers have a higher motivation to inhibit behavior and tend to prefer compromise, thus preferring a rounded shape. In addition, Zhu and Argo (2013) demonstrated that angular-shaped seating arrangements prime a need for uniqueness, while the rounded shaped prime a need to belong. Can this matching effect between shape and the need for uniqueness in seating arrangements be carried over into general research into shape preferences? Previous studies have shown that consumers with different power states will choose products that match their personal characteristics (Galinsky et al., 2015). Consumers experiencing a state of power are more likely to seek uniqueness, whereas those experiencing a state of powerlessness tend to seek conformity (Jin et al., 2011). Therefore, compared with low-power consumers, high-power consumers may have a higher need for uniqueness, and thus prefer angular (vs. rounded) shapes. The above argument preliminarily demonstrates possible alternative explanations of conflict resolution style and the need for uniqueness, but compared with perceived competence and warmth, whether they are the main factors affecting consumers' preference for angular and rounded shapes in different power states must be further empirically explored.

In the next section, we report the empirical tests of our hypotheses. Studies 1 and 2 test our basic hypothesis (H1) that consumers in high-power states indicate a more favorable attitude toward angular shapes through surveys and experiments. Studies 3 and 4 explore the mechanisms underlying the observed effects (H2) and excludes two possible alternative explanations. Study 5 further verifies the mediating effect of this research by manipulating the mediating variables.

## Study 1: Correlational Evidence

Study 1 provides an initial test of Hypothesis 1 that consumers in high- and low-power states respond differently to angular vs. rounded shapes. We measure consumers' chronic sense of power and predicted consumers with a higher sense of power would respond more positively to an angular shape. In contrast, consumers with a lower sense of power would respond more positively to rounded shapes. Study 1 consists of two sub-studies (Study 1a and Study 1b) verifying Hypothesis 1 with participants from different sources. Study 1a was predominantly American, while Study 1b was predominantly Chinese, to ensure consistency of results across cultures.

### Study 1a

#### Method

##### Sample

A total of 311 workers (155 females and 156 males; M_age_ = 34.06, *SD* = 10.83) from the Prolific participant pool took part in the online study. The number of participants was specified a priori based on power analysis in several pilot studies with samples from the same population. The study took ~2 min, and each participant received a $0.3 reward for completing the study.

##### Procedure

We first used the scale to measure participants' general sense of power (Anderson et al., 2012). The scale consists of eight statements (e.g., *I think I have a great deal of power*) and the participants need to rate the extent to which they agreed with each statement, using a 7-point Likert scale (1 = *strongly disagree* to 7 = *strongly agree*, α = 0.92). We then showed participants three pairs of images (see Appendix A). One pair of images contained simple shapes, and the other two pairs of images contained brand logos. Simple shapes were included in order to eliminate the interference of other complex factors, and brand logos were included in order to confirm the universality of our findings and provide empirical evidence for the subsequent design of actual products and brand logos. In each pair, there were two corresponding images: one with a circular shape and another with an angular shape. According to the prior test, for the three pairs of images in this study, the participants perceived the angularity of the angular graphics to be significantly higher than the rounded graphics (*ps* < 0.05). At the same time, there were no significant differences in the aesthetics of angular and rounded graphics in each pair (*ps* > 0.05). In addition, the order of presentation of each pair and the images within each pair were both randomized. In line with previous research (Zhang et al., 2006) on shape references, participants were asked to rate their preference between each pair of images (1 = I definitely prefer A to B, 7 = I definitely prefer B to A). Because the angular shape or rounded shape images were presented randomly in the position of A or B, we recoded this variable in such a way that a higher score indicated preference for an angular shape over a rounded shape.

We also collected participants' information about age and sex and analyzed them as control variables because they have been shown to covary with power and shape preference in previous studies (Lieven et al., 2015; Rucker et al., 2018). In addition, we measured participants' subjective social status (i.e., respect, admiration, and voluntary deference in the eyes of others; Magee and Galinsky, 2008). As for the measurement of subjective social status, consistent with prior research (Blader and Chen, 2012; Blader et al., 2016), participants rated the extent to which they agreed with three statements, “People respect me,” “People admire me,” and “People voluntary respect me,” using the 7-point Likert scale (1 = *strongly disagree* to 7 = *strongly agree*; α =0.89). Although power and social status are distinct constructs (Keltner et al., 2003; Blader and Chen, 2012), they tend to be correlated and can be associated with similar outcomes (Magee and Galinsky, 2008). Thus, to show the effects of power above and beyond that of social status, social status serves as a control variable in our analysis.

#### Results and Discussion

In support of Hypothesis 1, the statistical results of the simple shape show the general sense of power was significantly positively correlated with preference for an angular shape over a rounded shape (Simple shape: *r* = 0.12, *p* = 0.032), which was also confirmed by the analysis of brand logos (Logo 1: *r* = 0.16, *p* = 0.006; Logo 2: *r* = 0.13, *p* = 0.025). In addition, the combined data of the three experimental image pairs showed similar results, thereby providing further evidence to verify Hypothesis 1 (*r* = 0.19, *p* = 0.001). When controlling for participants' age, sex, and social status, power remained to be significantly positively related to the preference for an angular shape over a rounded shape (β = 0.19, *p* < 0.001). Specifically, Simple shape: β = 0.11, *p* = 0.046; Logo 1: β = 0.15, *p* = 0.005; Logo 2: β = 0.14, *p* = 0.012. Thus, participants with a higher sense of general power in their lives were more likely to prefer an angular shape over a rounded shape than those who had lower power.

Study 1a chose one simple shape and two different logo graphics as experimental materials for dependent variables and verified the influence of power state on shape preference. In addition to simple shapes and logos, angular and rounded design elements can also be reflected in products. Therefore, although the procedure of Study 1b is similar to that of Study 1a, there are two main considerations in the design of Study 1b; one is the replacement of experimental materials, and the other is the cross-cultural research of participants. In Study 1b, we chose two specific products, cushion and mug, as our experimental materials (See Appendix B) to test whether the power state will affect the preference of product shape. Additionally, the participants of Study 1b were Chinese.

### Study 1b

#### Method

##### Sample

A total of 233 adult consumers (132 females and 101 males; *M*_*age*_ = 21.84, *SD*_*age*_ = 5.31) participated in this study, and each participant received a ¥1 reward for completing the study.

##### Procedure

The procedure of Study 1b was consistent with that of Study 1a. Participants were asked to measure their sense of power, shape preference for the two pairs of images, and the demographic information in turn.

### Results and Discussion

The experimental results also support Hypothesis 1—general sense of power was significantly positively correlated with the preference for an angular shape over a rounded shape (Cushion: *r* = 0.13, *p* = 0.043; Mug: *r* = 0.17, *p* = 0.010). In addition, as for control variables, in Study 1a, we controlled for participants' age, sex, and social status, but in Study 1b, we only controlled for participants' sex and age because most participants in this study were students. In contrast to those who have been working, students have poor sensitivity to social status. When controlling for participants' age and sex, the positive influence of power on the preference for an angular shape was still significant (Cushion: β = 0.13, *p* = 0.047; Mug: β = 0.15, *p* = 0.021).

Studies 1a and 1b measured participants' sense of power as an individual differing factor, provided initial evidence for Hypothesis 1 that consumers experiencing higher power are more likely to prefer an angular shape over a rounded shape than consumers experiencing lower power. In addition, Studies 1a and 1b used different experimental materials and research objects. The above study design and statistical results show the conclusions of this study have good external validity. To better establish the causal relationship, in Study 2, instead of measuring the sense of power, we experimentally manipulated the high vs. low power state of participants to verify Hypothesis 1.

## Study 2: Experimental Evidence

Study 2 aimed to verify H1 through an experimental method. Study 2 also included two sub-studies (Study 2a and Study 2b) with participants from different countries. The participants in Study 2a were mainly American, while those in Study 2b were mainly Chinese.

### Study 2a

#### Method

##### Sample and Design

A total of 149 workers (61 females and 83 males; M_age_ = 33.11, *SD* = 9.00) were recruited from Amazon's Mechanical Turk to participate in this study. They were randomly assigned to the conditions of a single factor 3 (power: high vs. control vs. low) between-subjects design. The study took ~5 min, and each participant received a $0.6 reward for completing the study.

##### Procedure

We conducted the experiment as two unrelated studies. First, participants were asked to complete an episodic recall task (Galinsky et al., 2003; Rucker and Galinsky, 2008), frequently used in previous research to manipulate their power state. We asked participants in the high-power condition to recall a particular incident in which they had power over another individual and describe this situation in 5–7 sentences—namely what happened and how they felt. Participants in the low-power condition were asked to recall a particular incident in which someone else had power over them and describe this situation in 5–7 sentences—namely what happened and how they felt. While we asked participants in the control condition to recall a recent trip to a grocery store or convenience store, the requirements were the same as the other two conditions. Upon completion of the episodic recall task, participants were asked to rate the extent to which they feel “powerful, influential, important, powerless, subordinate, dependent” (α = 0.86) on 7-point scales as a power manipulation check. Items including powerless, subordinate, and dependent are reverse scored for the scale calculations. Subsequently, the participants were presented with a picture of a set of graphics (Logo 2; see Appendix C) and asked their preference for an angular shape over a rounded shape. The detailed measurement is the same as Study 1a. After completing the survey, the participants were debriefed and thanked.

#### Results

##### Manipulation Checks

The results of ANOVA showed that the power state was successfully manipulated [*F*_(2,146)_ = 33.88, *p* < 0.001]. Specifically, participants who were assigned to the high-power condition (*M* = 5.24, *SD* = 0.77) reported more powerful than those who were assigned to the low-power condition [*M* = 3.40, *SD* = 1.31; t_(92)_ = 8.45, *p* < 0.001] and control condition (*M* = 4.20, *SD* = 1.15; *t*_(105)_ = 5.47, *p* < 0.001). The degree of power reported by participants in the low-power condition was significantly lower than that in the control group [*t*_(95)_ = 3.17, *p* = 0.002).

##### Preference

An ANOVA performed on the preference score yielded a significant influence, *F*_(2,146)_ = 3.27, *p* = 0.041. Participants in the high-power condition (*M* = 5.44, *SD* = 1.72) reported more positive attitudes toward angular shape over rounded shape than those in the low-power condition [*M* = 4.45, *SD* = 2.35; *t*_(92)_ = 2.36, *p* = 0.021] and control condition [*M* = 4.65, *SD* = 2.04; *t*_(105)_ = 0.034], while the attitude between low-power condition and control condition was not significantly different [*t*_(95)_ = 0.45, *p* = 0.652].

### Study 2b

The procedure of Study 2b was similar to that of Study 2a, but with three major changes. First, instead of the online experiment, we conducted this study in the laboratory. Second, participants in this study were mainly Chinese. Third, to test the robustness of our effect, the experimental materials of the dependent variables in Study 2b were different from those of Study 2a.

#### Method

##### Sample and Design

A total of 166 undergraduate students from a public university participated in this study (89 females and 77 males; *M*_*age*_ = 19.89*, SD*_*age*_ = 3.46) and each participant received a ¥3 reward for completing the study. Participants were randomly assigned to one of the three conditions of a single factor 3 (power: high vs. control vs. low) between-subjects design.

##### Procedure

Participants completed the manipulation of the power state through an episodic recall task, after which, the sense of power scale (Anderson et al., 2012; α = 0.89) was used as a power manipulation check. Then, participants were presented with two pairs of images (one is the simple shape, and the other is the specific product mug; see Appendix D) to test their preference for angular shape over rounded shape.

#### Results and Discussion

##### Manipulation Checks

The results of the manipulation checks show that the power state was successfully manipulated, *F*_(2, 163)_ = 8.30, *p* < 0.001. Specifically, participants in the high-power condition (*M* = 4.50, *SD* = 1.27) reported more powerful than those in the low-power condition [*M* = 3.42, *SD* = 1.50; *t*_(105)_ = 4.04, *p* < 0.001] and control condition [*M* = 3.98, *SD* = 1.33; *t*_(112)_ = 2.08, *p* = 0.040). The degree of power reported by participants in the low-power condition was significantly lower than that in the control condition [*t*_(109)_ = 2.11, *p* = 0.037).

##### Preference

An ANOVA yielded a significant influence of power state on shape preference. For the simple shape, participants in the high-power condition (*M* = 4.58, *SD* = 1.63) reported more positive attitudes toward angular shape over rounded shape than those in the low-power condition [*M* = 3.81, *SD* = 1.66; *t*_(105)_ = 2.44, *p* = 0.017] and control conditions [*M* = 3.93, *SD* = 1.76; *t*_(112)_ = 2.04, *p* = 0.044], *F*_(2,163)_ = 3.32, *p* = 0.039. But the attitude between low-power condition and control condition has no significant difference [*t*_(109)_ = 0.38, *p* = 0.703]. Meanwhile, for the mug, the effect was consistent with the result of the simple figure. Participants in the high-power condition (*M* = 4.38, *SD* = 1.86) reported more positive attitudes toward angular shape over rounded shape than those in the low-power condition [*M* = 3.46, *SD* = 1.87; *t*_(105)_ = 2.55, *p* = 0.012] and control conditions [*M* = 3.56, *SD* = 1.97; *t*_(112)_ = 2.29, *p* = 0.024], *F*_(2,163)_ = 3.86, *p* = 0.023). The attitude between the low-power condition and control condition was not significantly different [*t*_(109)_ = 0.27, *p* = 0.790].

Studies 1 and 2 verified Hypothesis 1—consumers experiencing higher power are more likely to prefer an angular shape over a rounded shape than those experiencing lower power, demonstrated through different research methods, research objects, and experimental materials and improving the external validity of the conclusions. In Studies 3–5, we further tested the proposed mechanism that underlies the observed effect.

## Study 3: A Direct Test of Perceived Competence and Warmth

In order to better understand the relationship between power and shape preference, Study 3 mainly aimed to verify the mediating role of perceived competence and warmth, and to exclude alternative explanations of conflict resolution types and need for uniqueness.

### Method

#### Sample

A total of 288 workers (146 females and 140 males; *M*_*age*_ = 34.45, *SD*_*age*_ = 11.06) from the Prolific participant pool took part in the online study in exchange for a $0.3 reward.

#### Procedure

Just as in Study 1a, participants first rated the extent to which they agreed with the items of sense of power (Anderson et al., 2012), α = 0.91. To capture underlying mechanisms, participants were subsequently required to rate three items relating to the question, “When you look at an object, what characteristic do you value more about it?” Each item was rated on a scale from 1 to 7. The items were: (1) warmth vs. competence; (2) sincere vs. capable; (3) friendly vs. intelligent (Fiske et al., 2002). This was done to measure their valuing of competence and warmth (α = 0.79). Higher scores indicated higher values of competence. In addition, in order to exclude alternative explanations of the need for uniqueness and conflict resolution types, participants were then asked to fill in the scales of conflict resolution types (Morris et al., 1998; α = 0.83) and need for uniqueness (adapted from Tian and Mckenzie, 2001; α = 0.81). We then presented participants with an image of a pair of simple shapes (see Appendix E) and asked their preference for the angular and rounded shape (the same as Study 1a). Finally, the participants were debriefed and thanked.

### Results and Discussion

#### Preference

Results showed participants with a higher sense of power reported more positive attitudes toward angular shape over rounded shape (*r* = 0.14, *p* = 0.022), while controlling for participants' age, sex, and social status, the positive effect was still significant (β = 0.13, *p* = 0.024).

#### Mediation Analyses

**Figure 1:**
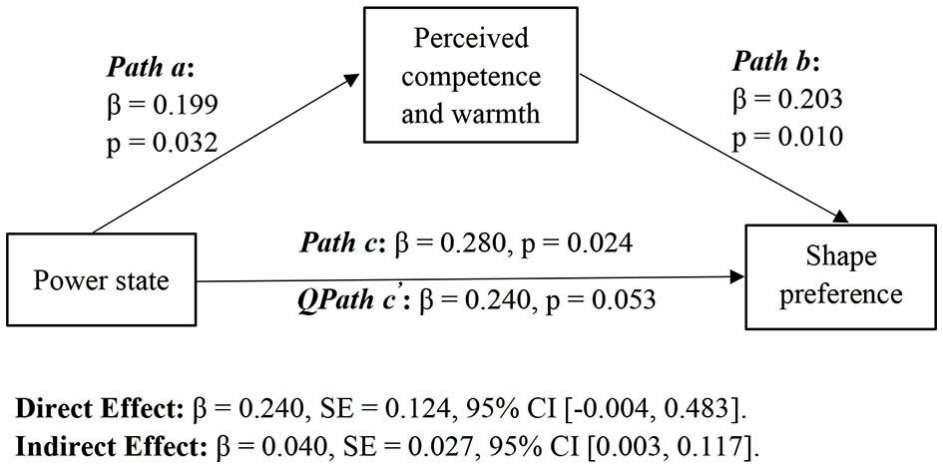
Mediation analyses. *p < 0.05, **p < 0.01, ***p < 0.001.

We performed a regression-based mediation analysis following Hayes' PROCESS macro (Hayes, 2013) to verify the mediating role of perceived competence and warmth. The results in Figure 1 show valuing competence over warmth mediated the positive relationship between experiencing higher power and the preference for an angular shape (indirect effect = 0.040, 95% bias-corrected CI [0.002, 0.118]). Meanwhile, we also examined the mediating effects of conflict resolution types and need for uniqueness. However, the results revealed that the mediation of conflict resolution types (indirect effect = 0.001, 95% bias-corrected CI [−0.012, 0.034]) and need to be unique (indirect effect = 0.007, 95% bias-corrected CI [−0.128, 0.058]) were not significant. The results of mediation analyses showed consumers experiencing higher power are reliant more on competence over warmth; thus, they are more likely to prefer an angular shape over a rounded shape than consumers experiencing lower power.

## Study 4: Experimental Evidence of Mediation

Study 3 preliminarily verified the mediating role of perceived competence and warmth through the survey method and indicated that, generally, consumers with high power pay more attention to competence characteristic and are thus more likely to prefer an angular shape, while low-power consumers pay more attention to warmth characteristic and are more likely to prefer a rounded shape. In Study 4, we used experimental manipulation to prime participants' sense of power in order to verify the causal relationship between sense of power and shape preference, and the mediating role of perceived competence and warmth.

### Method

#### Sample and Design

A total of 112 participants took part in this study (57 females and 55 males; *M*_*age*_ = 20.99, *SD*_*age*_ = 1.79) and each participant received a ¥3 reward for completing the study. Participants were randomly assigned to one of the two conditions of a single factor 2 (power: high vs. low) between-subjects design.

#### Procedure

Participants were asked to complete several unrelated tasks. First, we adopted procedures from Dubois et al. (2010) to manipulate participants' power state. Participants were randomly assigned the roles of manager (high-power condition) and employee (low-power condition). Participants in each condition were asked to read and complete a writing task. In the high-power condition, participants received the instructions, “Assume that you are the manager of the design department of an advertising company. You have complete control over coordination tasks, including work progress, the evaluation of subordinates, and the division of rewards. Then, as a leader, what are your feelings and thoughts, and how will you carry out your work and manage your subordinates?” In the low-power condition, participants received the instructions, “Assume that you are the employee of the design department of an advertising company. You have no control over the coordination task, and your performance, salary, and position promotion are judged by the leader. Then, as a subordinate, what are your feelings and thoughts, and how will you treat your work and your leader?” We again used the sense of power scale (Anderson et al., 2012) as a power manipulation check. The scale showed high reliability (α = 0.88).

Subsequently, just like Study 3, the participants were asked to rate in three items that contain the corresponding options: When you looked at an object what characteristic do you value more about it (1) 1 = pay more attention to warmth, 7 = pay more attention to competence; (2) 1 = pay more attention to sincere, 7 = pay more attention to capable; (3) 1 = pay more attention to friendly, 7 = pay more attention to intelligent (Fiske et al., 2002), to measure their valuing for competence and warmth (α =0.79). In addition, in order to exclude alternative explanations of the need for uniqueness and conflict resolution types, participants were then asked to fill in the scales of conflict resolution types (Morris et al., 1998; α =0.82) and need for uniqueness (adapted from Tian and Mckenzie, 2001; α =0.82). Then, participants were presented with three pairs of images (see Appendix F) to test their preference for angular shape over rounded shape. Finally, the participants were debriefed and thanked.

### Results and Discussion

#### Manipulation Checks

The results of the manipulation check showed that power state was successfully manipulated, *F*_(1, 110)_ =12.65, *p* =0.001. Specifically, participants in the high-power condition (*M* = 4.62, *SD* = 0.97) reported more powerful than those in the low-power condition (*M* = 3.90, *SD* = 1.17).

#### Preference

Combining participants' preference in the three pairs of images as the dependent variable, an ANOVA yielded a significant influence of power state on shape preference [*F*_(1,110)_ = 4.26, *p* = 0.041]. Specifically, participants in the high-power condition (*M* = 4.50, *SD* = 1.62) reported more positive attitudes toward angular shape over rounded shape than those in the low-power condition (*M* = 3.86, *SD* =1.65).

#### Mediation Analyses

**Figure 2:**
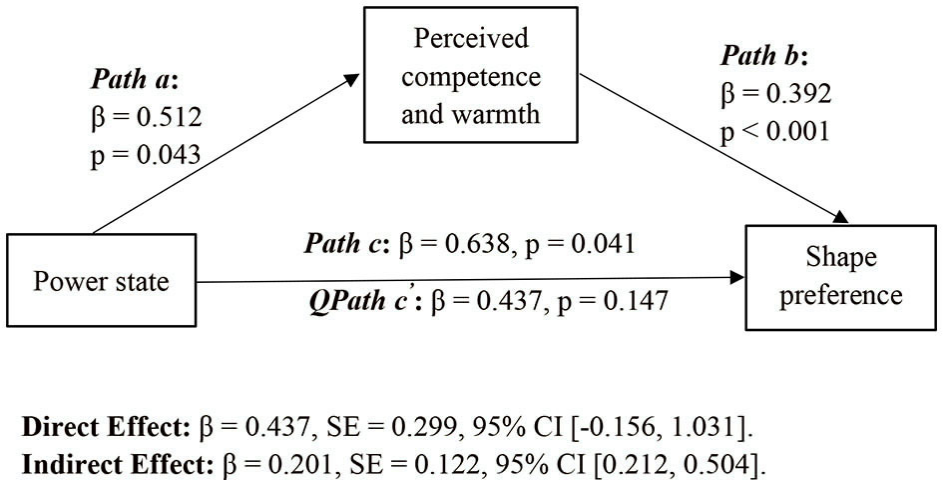
Mediation analyses. *p < 0.05, **p < 0.01, ***p < 0.001.

We conducted a mediation analysis with the participants in the high and low power conditions. In accordance with Hayes' (Hayes, 2013) study (model 4), we used a bootstrapping procedure that generated a sample size of 5,000 to assess the regression models. The results of this analysis indicated that participants with higher power valuing more on competence over warmth (β = 0.51, *t* = 2.05, *p* = 0.043), and the perceived competence has a positive impact on participants' preference toward angular shape over rounded shape (β = 0.39, *t* = 3.50, *p* < 0.001). Furthermore, the perceived competence and warmth mediated the positive relationship between power state and shape preference (indirect effect = 0.20, 95% bias-corrected CI [0.021, 0.504], direct effect =0.44, 95% bias-corrected CI [−0.156, 1.03]). Meanwhile, we also examined the mediating effects of conflict resolution types and need for uniqueness. However, the results revealed that the mediation of conflict resolution types (indirect effect =0.13, 95% bias-corrected CI [−0.010, 0.419]) and need to be unique (indirect effect =0.05, 95% bias-corrected CI [−0.048, 0.247]) were not significant. The results of mediation analyses showed high power consumers pay more attention on competence over warmth; thus, they are more likely to prefer an angular shape over a rounded shape than low power consumers (Figure 2).

## Study 5: Manipulate the Perceived Competence and Warmth

Study 3 and Study 4 verified the mediation effect through measurement and experiment respectively. However, sometimes, the product itself or its related advertising will also endow the product with certain competence and warmth attributes. Based on this, when designing a product, the most important subject is how to integrate shape design with product characteristics and product application context. Therefore, in Study 5, we verified the mediating mechanism again by manipulating the perception of competence and warmth in the laboratory. That is, when we manipulated the perceived competence and warmth attributes of marketing scenarios, whether the matching effect between consumers' power state and shape preference will still exist.

### Method

#### Sample and Design

A total of 246 undergraduates completed the study in the lab (134 females and 114 males; *M*_*age*_ = 19.25, *SD*_*age*_ = 1.12) and each participant received a ¥3 reward for completing the study. They were randomly assigned to one of the six conditions of a 2 (power states: low vs. high) * 3 (product attributes: competence vs. warmth vs. control) between-subjects design.

#### Procedure

Firstly, the participants completed the manipulation of the power state just like Study 4, after which, the sense of power scale (Anderson et al., 2012; α = 0.83) was used as a power manipulation check.

Subsequently, the participants in each condition were presented with an image of one pair of brand logos (see Appendix G, the order of presentation of angular shape and rounded shape was randomized), and informed that these are the pending logos of a certain automobile brand. Participants were asked to read the relevant introduction of the brand and choose the appropriate logo based on their understanding of the brand. In the competence condition, the product introduction emphasizes the performance advantages of the car. In the warmth condition, the product introduction emphasizes the warmth advantages of the car. In the control condition, the product introduction did not specifically emphasize any advantages. Specifically, participants in the competence condition received the information that “A company has recently developed a new type of car, which has strong power performance. The overall design adopts a high-tech and high configuration, with a strong driving force and excellent performance. At present, the company wants to create a simple and memorable brand logo for the car.” Participants in the warmth condition received the information that “A company has recently developed a new type of car, which is mainly aimed at the family car market, focusing on warmth and comfort. The design process fully reflects care and humanization. At present, the company wants to create a simple and memorable brand logo for the car.” Participants in the control condition received the instructions, “A company has recently developed a new type of car; at present, the company wants to create a simple and memorable brand logo for the car.” Then, the participants were asked to express their opinion on the suitability of the following two brand logos based on their understanding of the car (1 = A is very suitable, 7 = B is very suitable). Finally, the participants were debriefed and thanked.

### Results and Discussion

#### Manipulation Checks

An ANOVA performed on participants' sense of power yielded only a main effect of power such that the participants in the high-power condition reported more powerful (*M* = 4.15, *SD* = 1.49) than those in the low-power condition (*M* = 3.72, *SD* = 1.28), *F*_(1,246)_ = 5.85, *p* = 0.016. None of the other effects were significant (*p* > 0.28).

#### Preference

With participants' shape preference as the dependent variable, the ANOVA revealed significant main effects of power states [*F*_(1,242)_ = 6.39; *p* = 0.012] and product attributes [*F*_(2,242)_ = 34.81; *p* < 0.001). There was also a significant interaction between power states and product attributes [*F*_(2,242)_ = 3.09, *p* = 0.047). Specifically, in the control condition of product attributes, high-power participants reported higher preference (*M* = 4.71, *SD* = 1.60) toward angular shape over rounded shape than low-power participants (*M* = 3.61, *SD* = 1.43), *t*_(81)_ = 3.32, *p* = 0.001. Most importantly, compared with control conditions, in the competence condition, both high-power participants (*M* = 4.85, *SD* = 1.78) and low-power participants (*M* = 4.78, *SD* = 1.29) preferred an angular shape over rounded shape, and the difference between them was not significant [t_(80)_ = 0.21, *p* = 0.832]. On the contrary, in the warmth condition, both high-power participants (*M* = 3.07, *SD* = 1.23) and low-power participants (*M* = 2.86, *SD* = 1.26) preferred rounded shape over angular shape, and the difference between them was not significant [t_(81)_ = 0.79, *p* = 0.432].

The results of the experiment showed when the products' competence and warmth attributes were not manipulated (control condition), compared with low-power consumers, high-power consumers prefer an angular shape over a rounded shape, consistent with Hypothesis 1. When the products' competence and warmth attributes were manipulated, the effect of power states on shape preference disappeared, again verifying the mediating effect of competence and warmth (Hypothesis 2).

## General Discussion

In the current research, we conducted four studies and provided supportive evidence for the hypothesis that consumers in different power states respond differently to angular and rounded shapes. Study 1 provided preliminary evidence that consumers with a higher power state prefer an angular (vs. rounded) shape design than those with a lower power state. In Study 2, the causal relationship between them was further verified by an experimental method. Study 3 and Study 4 further verified the underlying mechanism of perceived competence and warmth; specifically, high-power consumers responded to the angular shape more positively because consumers experiencing higher power are valuated more on competence over warmth, thus they are more likely to prefer an angular shape over a rounded shape than consumers experiencing lower power. Study 5 provided further evidence of the underlying mechanism. That is, when we manipulated the perceived competence and warmth of the product attribute, the shape of the product or brand logo is integrated with its attributes; then, at this time, the matching effect between power state and shape preference disappears.

### Theoretical and Practical Implications

Our research contributes to the literature in several ways. First, the conclusions of this study expand the research of power state in the field of sensory marketing. Existing studies on power state mainly focus on persuasive messages (Dubois et al., 2016), compensatory consumption (Dubois et al., 2012), spending preferences (Rucker et al., 2011), and price fairness perceptions (Jin et al., 2014), but are not involved in the field of sensory marketing. However, sensory marketing has been a rapidly growing field in recent years (Krishna, 2012; Bajaj and Bond, 2018; Mookerjee et al., 2021) and has an important position in consumer behavior research. The current research, therefore, explores the preference effect of power state on visual shapes that have an important influence in sensory marketing, and proposes the matching effect between the two driven by the psychological propensities of power, which provides a new research idea for the power state and enriches the literature on power in the field of consumer behavior.

Second, as crucial visual elements, angular and rounded shapes have been widely studied by scholars and marketers (Bajaj and Bond, 2018; Vallen et al., 2018; Yang and Chen, 2019). However, the existing research on shape mainly focuses on the marketing effect brought about by shape design. For example, Yang and Chen (2019) demonstrated that in the context of precise numbers (vs. round numbers), the presentation of angular brand logos led to higher consumer evaluation than the presentation of rounded brand logos. However, studies on the factors that affect consumers' preference for different shapes are relatively scarce. Therefore, the current research attempts, from the perspective of psychology, to explore the relationship between power state and consumers' shape preference, enriching the literature of shape design and providing a new perspective for the study of sensory marketing.

Finally, a new underlying mechanism is introduced to explain the relationship between power and shape preference. The existing mechanism of studies on angular and rounded shapes focused on conflict resolution (Morris et al., 1998) and need for uniqueness (Tian and Mckenzie, 2001), which cannot adequately explain our current theory. In this paper, we introduce a new mediation mechanism, i.e., perceived competence and warmth. As two fundamental dimensions of social judgments, marketing scholars have widely studied perceived competence and warmth in recent years, and many important research conclusions have been obtained (Dubois et al., 2016). However, the existing research on competence and warmth mainly focuses on the perception and judgment of people or organizations but does not involve products or goods. Thus, this study initiates a new research field for scaling competence and warmth.

This study has several practical implications for advertisers and marketing professionals. First, our conclusions have important marketing implications for targeting different groups of customers. Specifically, the results show that high-power consumers prefer angular shape design, while low-power consumers prefer rounded shape design. Therefore, in practice, marketers can perform market segmentation based on variables correlated with the sense of power, such as socioeconomic status to design or recommend different products for consumers with different power states. Second, due to the scenario and maneuverability of power state, in the actual marketing strategy, marketers can provide advertising and environmental cues that shift consumers' current state of power according to their own product and brand characteristics, to achieve consistency in consumer power state and product characteristics, improving consumers' purchase intention and reducing their price sensitivity. Finally, graphic shapes are a very important part of marketing. This study points out that different shapes have different characteristics. In particular, the angular shape reflects more on competence, while rounded shape reflects more on warmth. According to this conclusion, businesses can design their brands and product shapes according to the marketing connotations they pursue to optimize the “self-expression” of products and obtain better consumer evaluations.

### Research Limitations and Future Research Directions

First, the influence of power state on consumers' thoughts and behaviors is mostly performed from the perspective of psychological propensities and psychological needs. This paper is based on the psychological propensities of power to explore the shape preferences of different power states. However, at present, the relevant research is based on a single perspective of psychological propensities or psychological needs. Through theoretical retrieval and experimental testing, we find that psychological propensities and psychological needs may jointly affect consumption behavior and their impact on individuals' consumption behavior may be different. Therefore, future research can follow this line of thinking to explore the different effects of power's psychological propensities and psychological needs impact on the same consumption phenomenon, which may lead to new findings.

Second, this paper explores the influence of power states on the preference for angular and rounded shapes, enriching the literature in the field of visual design. In the field of visual sense, in addition to shape, there are other visual elements. For example, Bajaj and Bond (2018) explored the relationship between the symmetry of graphics and brand personality. In future research, we will further explore the effect of regular and irregular graphics and complex and simple graphics. In addition to visual elements, the influence of color on consumer behavior has also gradually developed in recent years (Lee et al., 2016; Hagtvedt and Brasel, 2017), and new exploration in this field can also be performed in the future.

## Data Availability Statement

The datasets generated for this study are available on request to the corresponding author.

## Ethics Statement

Ethical review and approval were not required for this type of study on human participants in accordance with the local legislation and institutional requirements. The patients/participants provided their written informed consent to participate in this study.

## Author Contributions

FY designed the study, analyzed and interpreted the data, conceived and drafted the manuscript. XJ participated in the interpretation of results and edited the manuscript. BW provided important reference opinions for research design, and critically revised the manuscript. TZ conducted data collection and conducted writing instruction. TM co-designed the study, and participated in the data collection. All authors contributed to the article and approved the submitted version.

## Funding

The current study was supported by the National Natural Science Foundation of China (Grant 71872070, 71902069, 71902148), Social Science Foundation project of Jilin Province (Grant 2020C060), China Postdoctoral Science Foundation (Grant 2019M660243), and Sichuan University (Grant 2020CXQ23).

## Conflict of Interest

The authors declare that the research was conducted in the absence of any commercial or financial relationships that could be construed as a potential conflict of interest.

## Publisher's Note

All claims expressed in this article are solely those of the authors and do not necessarily represent those of their affiliated organizations, or those of the publisher, the editors and the reviewers. Any product that may be evaluated in this article, or claim that may be made by its manufacturer, is not guaranteed or endorsed by the publisher.
