# Supplementary material for: Shape–Trait Consistency: The Matching Effect of Consumer Power State and Shape Preference
Source: Front Psychol. 2021 Sep 30;12:615647. doi: 10.3389/fpsyg.2021.615647 (PMC8514985; doi:10.3389/fpsyg.2021.615647)
Supplement: Supplementary file 1 [file Data_Sheet_1.docx]

**Appendix**

Appendix A

*The order of presentation of each pair and that of the images within each pair were both randomized*

| Simple shape | 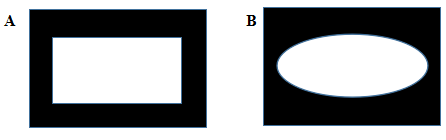 | 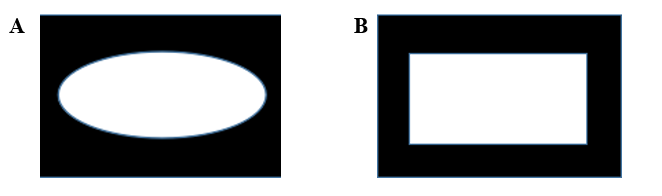 |
| --- | --- | --- |
| Logo1 | 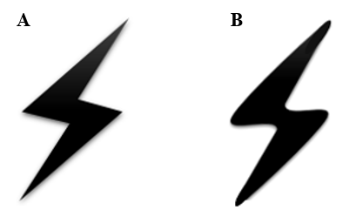 | 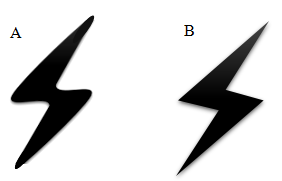 |
| Logo2 | 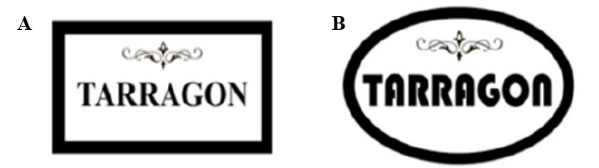 | 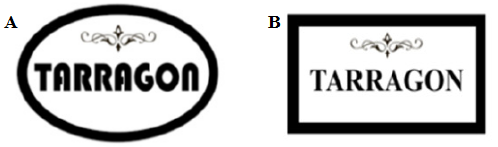 |

Appendix B

*The order of presentation of each pair and that of the images within each pair were both randomized*

| Cushion | 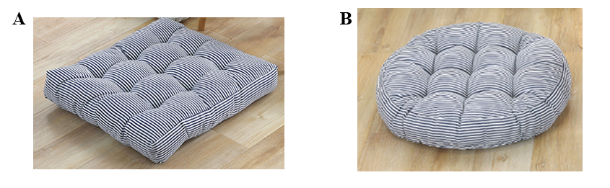 | 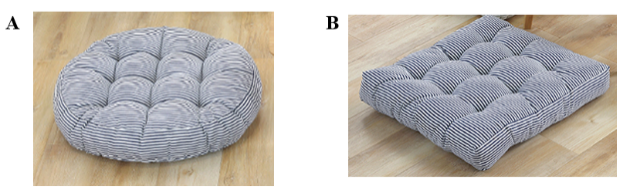 |
| --- | --- | --- |
| Mug | 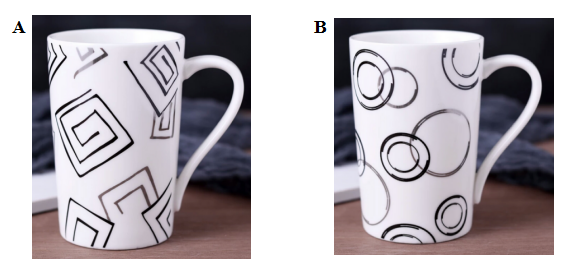 | 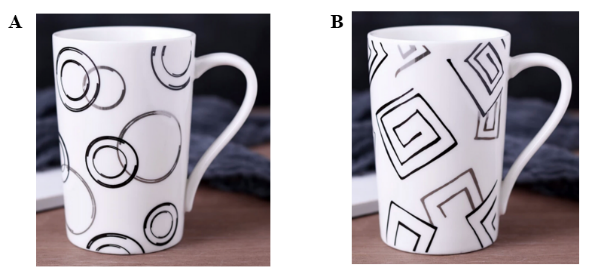 |

Appendix C

*The order of presentation of each pair and that of the images within each pair were both randomized*

| Logo 2 | 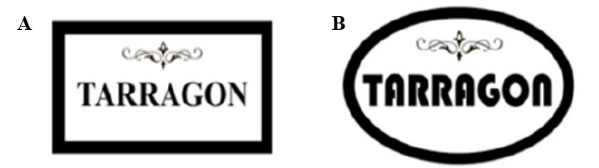 | 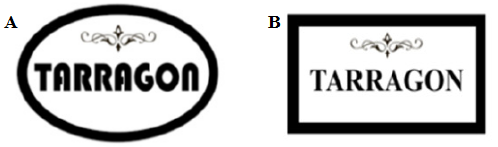 |
| --- | --- | --- |

Appendix D

*The order of presentation of each pair and that of the images within each pair were both randomized*

| Simple shape | 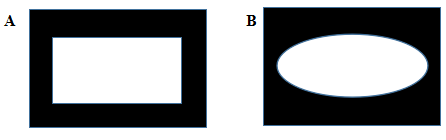 | 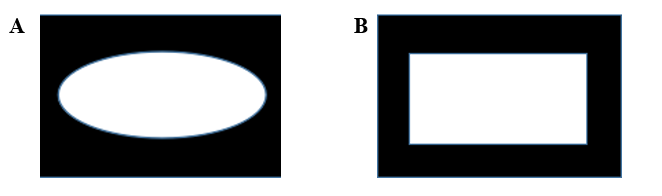 |
| --- | --- | --- |
| mug | 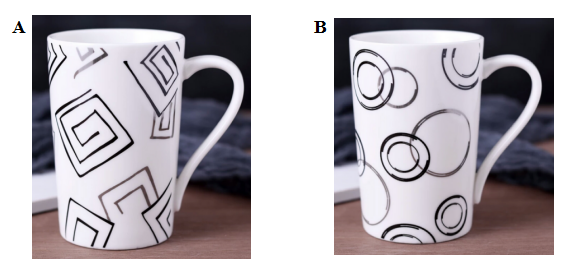 | 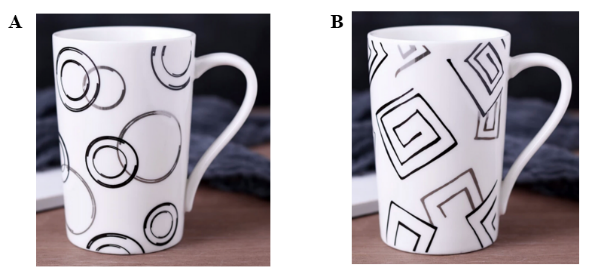 |

Appendix E

*The order of presentation of each pair and that of the images within each pair were both randomized*

| Simple shape | 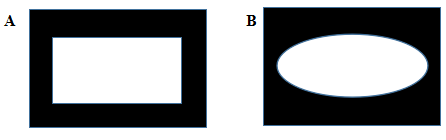 | 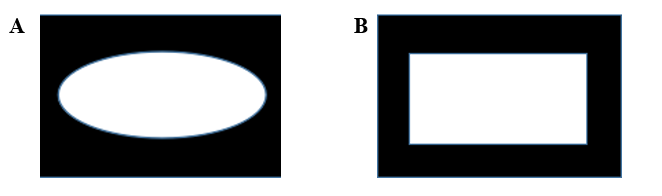 |
| --- | --- | --- |

Appendix F

*The order of presentation of each pair and that of the images within each pair were both randomized*

| Simple shape | 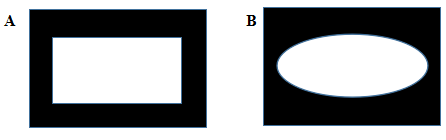 | 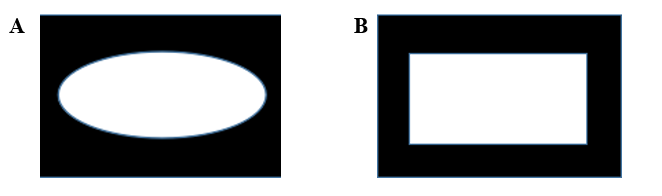 |
| --- | --- | --- |
| Logo1 | 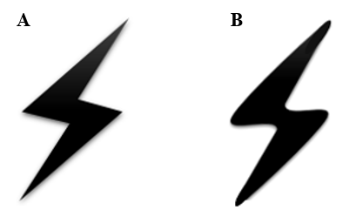 | 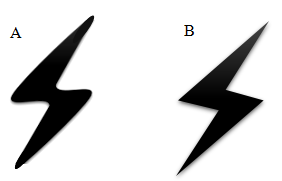 |
| Logo2 | 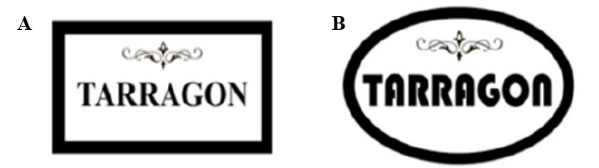 | 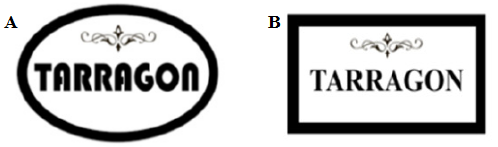 |

Appendix G

*The order of presentation of each pair and that of the images within each pair were both randomized*

| Logo1 | 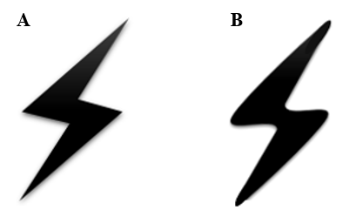 | 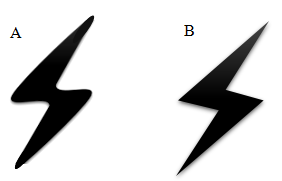 |
| --- | --- | --- |
